# Fibroblast growth factor-23 is associated with imaging markers of diabetic cardiomyopathy and anti-diabetic therapeutics

**DOI:** 10.1186/s12933-020-01135-z

**Published:** 2020-09-30

**Authors:** Martin H. Sørensen, Annemie S. Bojer, Niklas R. Jørgensen, David A. Broadbent, Sven Plein, Per L. Madsen, Peter Gæde

**Affiliations:** 1 Department of Cardiology and Endocrinology, Slagelse Hospital, Ingemannsvej 32, 4200 Slagelse, Region Zealand Denmark; 2 Institute of Regional Health Research, Faculty of Health Sciences, University of Southern Denmark, Odense, Denmark; 3 Department of Clinical Biochemistry, Rigshospitalet, Glostrup, Capital Region of Denmark Denmark; 4 Department of Medical Physics and Engineering, Leeds Teaching Hospitals NHS Trust, Leeds, UK; 5 Leeds Institute of Cardiovascular and Metabolic Medicine, University of Leeds, Leeds, UK; 6 Department of Cardiology, Copenhagen University Hospital Herlev-Gentofte, Hellerup, Capital Region of Denmark Denmark; 7 Department of Clinical Medicine, University of Copenhagen, Copenhagen, Denmark

**Keywords:** Type 2 diabetes, Myocardial perfusion, Fibroblast growth factor-23, Diabetic cardiomyopathy, Cardiac magnetic resonance imaging

## Abstract

**Background:**

The biomarker fibroblast growth factor-23 (FGF-23) has been associated with increased cardiovascular morbidity and mortality in both patients with and without type 2 diabetes. The aim of this study was to evaluate the relationship between FGF-23 and cardiac structure, function and perfusion in patients with type 2 diabetes and normal or mildly impaired kidney function. Furthermore, to investigate the association between FGF-23, anti-diabetes therapy and the classic complications and risk factors associated with type 2 diabetes.

**Methods:**

In this cross-sectional study, 246 patients with type 2 diabetes underwent echocardiography and advanced cardiac magnetic resonance imaging to assess left ventricular (LV) structure and function. In addition, myocardial blood flow (MBF) during rest and pharmacological stress (adenosine 140 µg/kg/min) were evaluated in 183 of the patients. Patients with eGFR < 60 ml/min/1.73 m^2^ were excluded.

**Results:**

Median (Q1–Q3) FGF-23 was 74 (58–91) ng/L. Patients with FGF-23 above the median had lower MBF during stress (2.3 ± 0.9 vs. 2.7 ± 0.9 ml/min/g, P = 0.001) and lower overall myocardial perfusion reserve (MPR) (2.7 ± 0.8 vs. 3.3 ± 1.1, P < 0.001). LV mass (143 ± 40 vs. 138 ± 36 g, P = 0.04) and E/e* (8.5 ± 3.2 vs. 7.6 ± 2.7, P = 0.04) were higher in patients with FGF-23 above the median. In a linear model adjusted for age, sex, eGFR and hypertension, increasing FGF-23 was associated with decreased MPR (P < 0.01, R^2^ = 0.11) and increased E/e* (P < 0.01, R^2^ = 0.07). FGF-23 was lower in patients receiving glucagon like peptide-1 (GLP-1) analogues (71 (57–86) vs. 80 (60–98) ng/L, P = 0.01) than in those who did not receive GLP-1 analogues.

**Conclusions:**

In patients with type 2 diabetes and normal or mildly impaired kidney function, increased levels of FGF-23 are associated with impaired cardiac diastolic function and decreased MPR, caused by a decrease in maximal MBF during stress. Use of GLP-1 analogues is associated with decreased levels of FGF-23.

*Clinical trial registration* https://www.clinicaltrials.gov. Unique identifier: NCT02684331. Date of registration: February 18, 2016

## Background

The link between type 2 diabetes and cardiovascular disease is well established. In patients with type 2 diabetes, the risk of developing coronary artery disease (CAD), heart failure, and cardiovascular death is significantly increased compared to those without type 2 diabetes [1, 2]. The mechanism and pathophysiology behind this increased risk of cardiovascular disease have been of interest for researchers for decades; yet, no conclusive results or methods for identifying those at increased risk have been discovered so far. Fibroblast growth factor-23 (FGF-23) is a hormone secreted from osteocytes and plays an important role in vitamin D and phosphate homeostasis [3]. When renal function declines in patients with chronic kidney disease the concentration of circulating FGF-23 is increased in response to persistent hyperphosphatemia [4]; however, increased levels of FGF-23 have also been associated with an increased risk of developing heart failure, cardiovascular disease, and cardiovascular death independent of renal function and other cardiovascular risk factors [5, 6]. In patients with combined type 2 diabetes and CAD, FGF-23 independently predicts adverse cardiovascular outcome [7] and, additionally, elevated FGF-23 concentrations following acute coronary syndrome are associated with an increased risk of CV death and hospitalization due to heart failure [8]. Furthermore, a recent study of patients with type 2 diabetes and normal or mildly impaired kidney function showed that FGF-23 was associated with an increased risk of both major adverse cardiovascular events and all-cause mortality [9]. Given the evidence of a relationship between levels of FGF-23 and cardiovascular outcome, we hypothesized that FGF-23 may be associated with structural and functional changes in the heart. Previous research on this topic is scarce and, to our knowledge, it has never been investigated using advanced imaging techniques such as cardiac magnetic resonance imaging (CMR), a technique that allows for detailed and accurate assessment of cardiac structure, function and perfusion. In this study, we investigated the association between FGF-23 and cardiac remodeling and subclinical cardiac disease measured by CMR in a large cohort of patients with type 2 diabetes and normal or mildly impaired kidney function. In addition, we investigated the relationship between FGF-23, anti-diabetes therapy and the classic complications and risk factors known to be associated with type 2 diabetes.

## Methods

The study was conducted at the Department of Cardiology and Endocrinology, Slagelse Hospital, and at the Department of Radiology, Næstved Hospital, Denmark. It was approved by the local ethics committee, Region Zealand, Denmark (SJ-490) and complied with the declaration of Helsinki. Detailed information about the study design and study cohort has previously been published [10]. In short, this study is part of a cross-sectional survey of patients with type 2 diabetes. Patients were recruited from the outpatient clinic at the Department of Endocrinology at Næstved/Slagelse/Ringsted (NSR) Hospitals, in the period from February 2016 to January 2019. Patients aged 18–80 years and with type 2 diabetes for at least 3 months were eligible to participate. Patients with permanent or persistent atrial fibrillation, contraindications to CMR or an eGFR < 30 ml/min/1.73 m^2^ (contraindication to the contrast agent gadolinium) were excluded from the original study cohort. In patients with chronic kidney disease, FGF-23 can rise to very high levels. To reduce the risk of confounding, we further excluded patients with eGFR < 60 ml/min/1.73 m^2^ in this study, and only examined those with normal or mildly impaired kidney function. Prior to participation, all patients gave written informed consent. All patients underwent clinical examination including evaluation of diabetes complication status, echocardiography, CMR and blood- and urinary sampling. Information regarding patient medication, history of smoking, coronary artery disease, hypertension and duration of diabetes were collected from the electronical patient journal or reported by the patients themselves. Hypertension was defined as clinical blood pressure > 140/90 mmHg after 10 min of rest or as an active prescription of antihypertensive medication. Coronary artery disease was defined as angiographically verified coronary stenosis, previous myocardial infarction/percutaneous coronary intervention/coronary artery bypass graft or ischemic lesions on late gadolinium enhancement (LGE) CMR. Albuminuria was defined as a urinary albumin/creatinine ratio (UACR) > 30 mg/g and retinopathy was evaluated from the patients’ latest fundus photography, routinely performed in the diabetes outpatient clinic. Autonomic nervous function was evaluated using orthostatic blood pressure measurements and beat-to-beat variation. Autonomic neuropathy was diagnosed in patients with a decrease of 25 mmHg or more in the orthostatic systolic blood pressure measurement [11] or with beat-to-beat variation < 4 beats/min [12]. Peripheral neuropathy was evaluated from the patient’s latest chiropodist report and diagnosed in patients whose report indicated signs of sensory nerve damage, patients with typical symptoms of peripheral neuropathy and in male patients with erectile dysfunction. Patients were stratified as receiving mono-, dual- or combination therapy based on their anti-diabetes medication (one, two or three or more anti-diabetes drugs, respectively).

### Echocardiography and CMR protocol

2D Echocardiography was performed on a GE healthcare Vivid E9 cardiovascular ultrasound system, using a GE Vivid S5 probe. CMR was performed on a 1.5 T scanner (Siemens Avanto; Erlangen, Germany) with spine- and cardiac coil, electrocardiographic gating and patients in the supine position. Detailed information about image acquisition and CMR sequence parameters has previously been published [10]. Left ventricular (LV) diastolic function was evaluated from echocardiography. In the apical four-chamber view, peak E was defined as the highest early mitral inflow velocity measured by pulse-wave Doppler. Septal and lateral mitral annular velocity (e*) were measured using tissue Doppler. E/e* was calculated as the mean of septal and lateral e* values. When taken individually, E/e* has shown the best correlation with invasive measurements of elevated LV filling pressure [13]. Left atrial (LA) structure and LV structure and systolic function were evaluated with CMR. Myocardial perfusion was obtained from 3 short-axis slices (basal, mid-ventricular and apical level) during rest and pharmacological stress (adenosine infusion at a dose of 140 µg/kg/min for 3 min) using a saturation recovery pulse sequence with spoiled gradient echo readout. A contrast dose of 0.075 mg/kg of gadobutrol (Gadovist®, Bayer AG, Germany) was administered at a rate of 5 ml/s followed by 20 ml of saline for both rest and stress perfusion scans. T_1_ maps were acquired at similar positions as the perfusion sequences, using a breath-held Shortened Modified Look-Locker Inversion (ShMOLLI) recovery sequence. Native T_1_ mapping was performed prior to the stress perfusion scan; post-contrast T_1_ mapping was performed 10 min after contrast injection and just before the rest perfusion scan. LGE images were acquired using a phase sensitive inversion recovery reconstruction sequence. LGE images were acquired from the entire LV short axis stack and from the two-, three- and four-chamber views LGE lesions were considered positive if present in 2 or more views.

### CMR data analysis

CMR scans were analyzed with cmr42® (Circle Cardiovascular Imaging Inc., Calgary Canada, v. 5.5.1). LV ejection fraction (LVEF), LV end-diastolic volume (EDV), LV end-systolic volume (ESV), LV stroke volume (SV), LV ejection fraction (LVEF), LV mass and LA max volume were analyzed as previously described [14]. Quantification of myocardial blood flow was performed for the mid-slice perfusion data using an in-house tool developed in MATLAB 2015b (MathWorks, Nattick, MA, USA). Perfusion images were segmented based on the American Heart Association 16-segment model [15] and additional regions were drawn in the LV blood pool in both the perfusion images and T_1_ map. The non-linear response of signal intensity to contrast agent concentration was corrected for based on the baseline signal intensity and T_1_ data [16]. Data were cropped to the end of the first-pass and Fermi-constrained deconvolution [17] was performed to yield segmental myocardial blood flow (MBF) estimates. Myocardial extracellular volume fraction (ECV), a variable shown to correlate well with diffuse myocardial fibrosis [18], was calculated from native and postcontrast myocardial T_1_ times and hematocrit values as previously described [19]. ECV was calculated as the mean value of the 6 midventricular segments and any areas with infarction seen on LGE images were excluded.

### Fibroblast growth factor-23

Patients had blood sampling performed just prior to the CMR scan. Following initial handling, the blood samples were immediately transferred to 1 ml cryotubes and stored at −80 °C for future usage. After a single freeze–thaw cycle, FGF-23 was analyzed on EDTA-plasma samples using a chemiluminescence immunoassay (CLIA) on a fully automated chemiluminescence analyzer, the Liaison XL (Diasorin, Saluggia, Italy).

### Statistical analysis

Normally distributed continuous variables are expressed as mean ± SD and categorical variables as percentages. Skewed variables are expressed as median and interquartile range (IQR). Depending on the distribution, continuous variables were compared using an unpaired two-tailed student’s t-test or Mann–Whitney U test, and categorical variables were compared using the χ^2^-test or Fisher’s exact test, as appropriate. The general linear model was used for analysis of explanatory variables. Distribution of FGF-23 was skewed and logarithmically transformed for linear correlation. A two-tailed P-value < 0.05 was considered statistically significant. Statistical calculations were made in SAS Enterprise Guide v. 7.15 (SAS Institute inc., Cary, NC, USA).

## Results

In total, 273 patients with available FGF-23 measurements were included in the original study cohort. Twenty-seven patients with eGFR < 60 ml/min/1.73 m^2^ were excluded, leaving 246 patients in the final study cohort. Myocardial perfusion was measured in 183 of the 246 patients. In the entire cohort, FGF-23 ranged from 18.9 to 219.4 ng/L with a median level of 74 (IQR 58–91) ng/L.

**Fig. 1:**
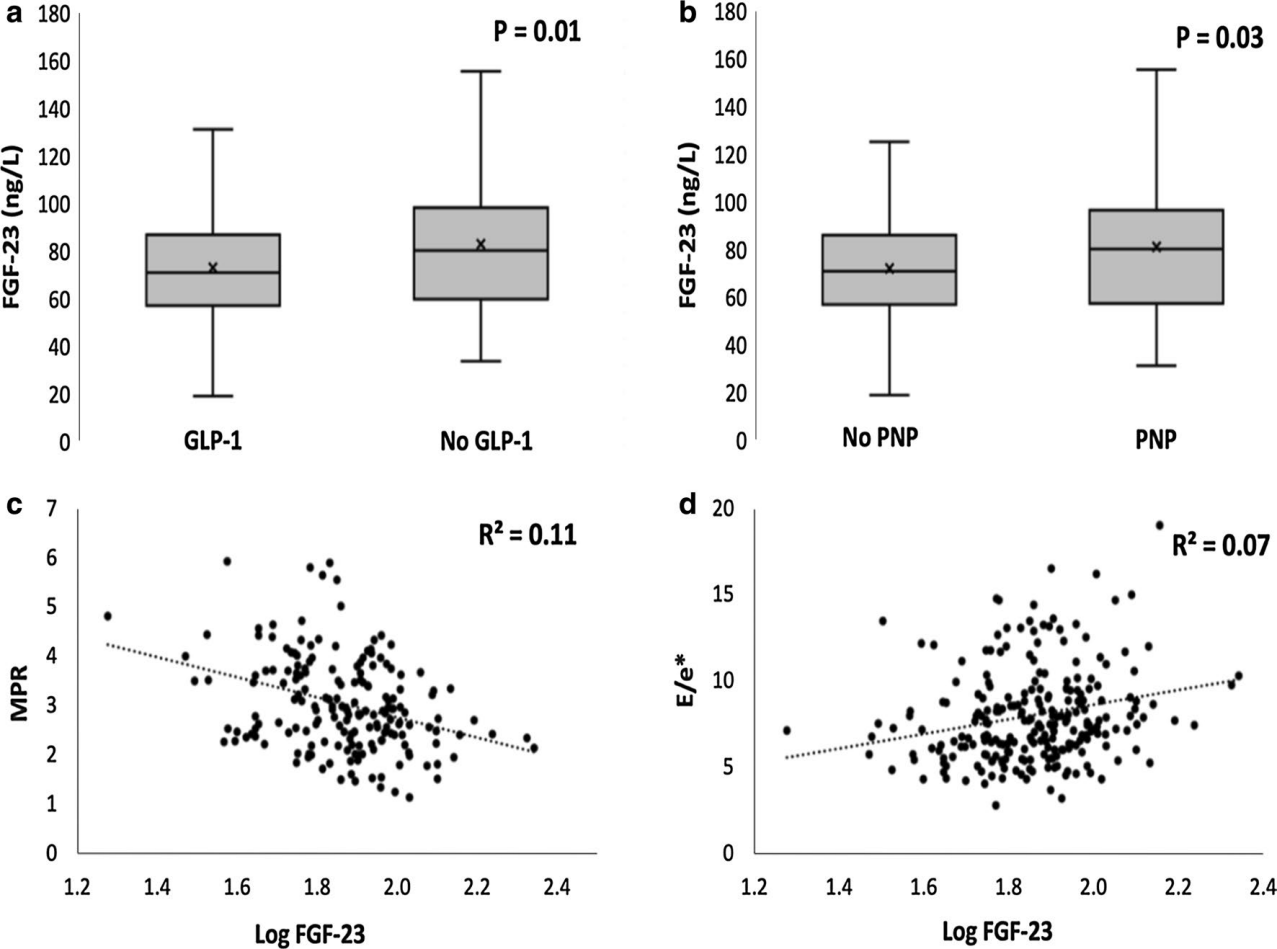
FGF-23 in patients receiving treatment with GLP-1 analogues alone or in combination with other anti-diabetes medication compared to those who did not receive treatment with GLP-1 analogues (**a**). FGF-23 in patients with and without peripheral neuropathy (**b**). Correlation between FGF-23 and myocardial perfusion reserve (MPR) (**c**) and FGF-23 and E/e* as a measure of cardiac diastolic function (**d**)

Clinical characteristics of patients with FGF-23 below and above the median are presented in Table 1. Peripheral neuropathy and hypertension were more prevalent in patients with FGF-23 above the median. Angiotensin converting enzyme inhibitors/angiotensin II-receptor blockers (ACE-i/ARB) were more frequently used in patients above the median whereas patients with FGF-23 below the median had higher usage of glucagon-like peptide 1 (GLP-1) analogues than did patients above the median. Levels of biomarkers associated with cardiac remodeling and cardiac myocyte damage (proANP, proBNP, hs-TnT and hs-CRP) did not differ between patients with FGF-23 below and above the median. When directly compared, FGF-23 levels were lower in patients treated with GLP-1 analogues than in those who were not treated with GLP-1 analogues (71 (57–86) vs. 80 (60–98) ng/L, P = 0.01) (Fig. 1a) and in patients without signs of peripheral neuropathy compared to those with peripheral neuropathy (70 (57–85) vs. 80 (57–97) ng/L, P = 0.03) (Fig. 1b). FGF-23 in relation to different combinations of anti-diabetes medication is presented in Table 2.

**Table 1:** Clinical characteristics of patients with FGF-23 below and above the median

	Fibroblast growth factor-23		P-value
	Below the median	Above the median	
Age (years)	58 ± 11	59 ± 10	0.28
Male sex	84 (68)	89 (72)	0.49
BMI (kg/m^2^)	30.7 ± 4.7	31.8 ± 4.4	0.06
Duration of diabetes (years)	12 ± 8	13 ± 8	0.14
Albuminuria	37 (30)	51 (41)	0.08
Retinopathy	39 (32)	32 (26)	0.32
Autonomic neuropathy	27 (22)	36 (29)	0.19
Peripheral neuropathy	43 (35)	60 (49)	**0.03**
Clinical SBP (mmHg)	136 ± 15	139 ± 18	0.12
Resting HR (bpm)	72 ± 13	72 ± 11	0.94
Coronary artery disease	18 (15)	22 (18)	0.49
Hypertension	72 (59)	97 (79)	** < 0.001**
Active or former smoking	81 (66)	85 (69)	0.53
HbA1c (% [mmol/mol])	7.8 ± 1.4 (62 ± 15)	7.9 ± 1.3 (63 ± 14)	0.56
eGFR (ml/min/1.73 m^2^)	85 ± 5	82 ± 10	**0.002**
LDL cholesterol (mmol/L)	2.1 ± 0.9	1.9 ± 0.8	0.06
HDL cholesterol (mmol/L)	1.2 ± 0.4	1.2 ± 0.3	0.31
Total cholesterol (mmol/L)	4.4 ± 1.2	4.2 ± 0.9	0.07
proANP (pmol/L)	56 (41–97)	59 (44–105)	0.45
NT-proBNP (pmol/L)	5.9 (5.9–10.8)	5.9 (5.9–11.0)	0.71
Hs-TnT (ng/L)	13 (13–15)	13 (13–17)	0.38
Hs-CRP (mg/L)	2.0 (1.0–4–6)	2.3 (1.1–4.3)	0.72
Urine ACR (mg/mmol)	16 (8–42)	18 (8–60)	0.23
*Medication*			
ACE inhibitor/ARB	80 (65)	98 (80)	**0.01**
Statins	79 (64)	87 (71)	0.28
SGLT-2 inhibitor	43 (35)	39 (32)	0.59
GLP-1 analogue	54 (44)	33 (27)	**0.005**
Insulin	77 (63)	69 (56)	0.30
Monotherapy	22 (18)	14 (11)	0.21
Dual therapy	45 (47)	46 (37)	0.89
Combination therapy	54 (44)	62 (50)	0.31

Data presented as mean ± SD, median (Q1–Q3) or as *n* (%). *ACR* albumin creatinine ratio, *ARB* angiotensin receptor blocker, *BMI* body mass index, *eGFR* estimated glomerular filtration rate, *GLP-1* glucagon like peptide 1, *HR* heart rate, *Hs-CRP* high sensitivity C-reactive protein, *Hs-TnT* high sensitivity troponin T, *NT-proBNP* N-terminal brain natriuretic peptide, *proANP* atrial natriuretic peptide, *SBP* systolic blood pressure, *SGLT-2* sodium-glucose cotransporter 2. Significant differences indicated in bold.

**Table 2:** Relations between FGF-23 concentrations and different combinations of anti-diabetes therapy

	Yes	No	P-value
SGLT-2 inhibitor only (n = 27)	71 (61–94)	75 (58–91)	0.89
GLP-1 analogue only (n = 20)	68 (55–87)	81 (60–98)	0.04*
SGLT-2 inhibitor + GLP-1 analogue (n = 16)	74 (57–91)	74 (61–92)	0.86
Insulin only (n = 67)	68 (55–86)	75 (60–94)	**0.03**
Insulin + SGLT-2 inhibitor (n = 28)	75 (62–90)	74 (57–91)	0.62
Insulin + GLP-1 analogue (n = 40)	72 (58–88)	83 (60–99)	**0.04**
Insulin + SGLT-2 inhibitor + GLP-1 analogue (n = 11)	74 (58–93)	75 (58–91)	0.76

Data presented as median (Q1–Q3). *GLP-1* glucagon like peptide-1, *SGLT-2* sodium-glucose cotransporter 2. Significant differences indicated in bold.

FGF-23 was significantly lower in patients treated with both GLP-1 analogues and insulin only or as combination therapy. Treatment with sodium-glucose cotransporter 2 (SGLT-2) inhibitors as monotherapy or in combination with GLP-1 analogues or insulin were not associated with any significant changes in FGF-23 concentrations. Table 3 shows cardiac structure, function and perfusion in patients with FGF-23 below and above the median. Patients with FGF-23 above the median had higher LV mass (143 ± 40 vs. 138 ± 36 g, P = 0.04) and average E/e* (8.5 ± 3.2 vs. 7.6 ± 2.7, P = 0.04) as well as lower MBF during stress (2.3 ± 0.9 vs. 2.7 ± 0.9 ml/min/g, P = 0.001) and lower myocardial perfusion reserve (MPR) (2.7 ± 0.8 vs. 3.3 ± 1.1, P < 0.001) compared with patients with FGF-23 below the median. LV mass remained significantly different after indexing to body surface area. We found no differences in LVEF, EDV, ESV, SV, LA max volume, rest MBF or ECV between patients with FGF-23 below or above the median.

**Table 3:** Relations between median levels of FGF-23 and measurements of cardiac structure, function and perfusion

	Fibroblast growth factor-23		P-value
	Below the median	Above the median	
LVEF (%)	63 ± 8	63 ± 7	0.92
EDV (ml)	153 ± 35	154 ± 37	0.91
EDV index (ml/m^2^)	72 ± 13	70 ± 15	0.29
ESV (ml)	58 ± 22	59 ± 24	0.75
ESV index (ml/m^2^)	27 ± 9	27 ± 10	0.78
SV (ml)	95 ± 21	95 ± 20	0.89
LV mass (g)	138 ± 36	143 ± 40	**0.04**
LV mass index (g/m^2^)	62 ± 13	66 ± 16	**0.03**
LA volume (ml)	91 ± 25	97 ± 27	0.07
LA volume index (ml/m^2^)	41 ± 11	44 ± 11	0.10
Rest MBF (ml/min/g)	0.81 ± 0.19	0.83 ± 0.18	0.69
Stress MBF (ml/min/g)	2.7 ± 0.9	2.3 ± 0.9	**0.001**
MPR	3.3 ± 1.1	2.7 ± 0.8	** < 0.001**
Average E/e*	7.6 ± 2.7	8.5 ± 3.2	**0.04**
Extracellular volume (%)	27.8 ± 3.5	28.6 ± 3.2	0.51

Data presented as mean ± SD. *LVEF* left ventricular ejection fraction, *EDV* end-diastolic volume, *ESV* end-systolic volume, *SV* stroke volume, *LA* left atrial, *MBF* myocardial blood flow, *MPR* myocardial perfusion reserve. Significant differences indicated in bold.

In a linear model, increasing FGF-23 was associated with decreased MPR (R^2^ = 0.11, P < 0.001) (Fig. 1c), increased E/e* as a measure of cardiac diastolic function (R^2^ = 0.07, P = 0.001) (Fig. 1d) and increased urinary albumin creatinine ratio (R^2^ = 0.05, P = 0.02). MPR and E/e* remained significantly associated with FGF-23 when the model was adjusted for age, sex, eGFR and known hypertension (P < 0.01 for both), whereas UACR lost its significance after adjustment. LV mass was not significantly associated with FGF-23 in a linear model.

## Discussion

An independent relationship between increased circulating levels of FGF-23, incident heart failure and cardiovascular mortality has previously been described in large community-based studies [5, 6] and, recently, a similar relationship was confirmed in a larger cohort of patients with type 2 diabetes [9]. It has not previously been systematically investigated, whether increased concentrations of FGF-23 in patients with type 2 diabetes are associated with changes in cardiac function and morphology, as a possible mechanistic explanation for the increased prevalence of cardiovascular events seen in this patient group. In this study, we examined a cohort of patients with type 2 diabetes using echocardiography and quantitative cardiac magnetic resonance imaging, and found that increased levels of FGF-23 were associated with reduced MPR, caused by a decrease in maximal MBF during pharmacological stress, and signs of impaired diastolic function.

### FGF-23 and myocardial perfusion

In the past decades, there has been an emerging interest in diabetic cardiomyopathy and non-ischemic heart failure in patients with type 2 diabetes, and a link between myocardial microvascular disease and cardiac function has previously been demonstrated and suggested as an underlying mechanism of diabetic cardiomyopathy [10, 14, 20]. We found that increasing levels of FGF-23 were associated with a decrease in MPR due to impaired perfusion during vasodilator-induced stress, further supporting the theory that microvascular disease plays an important role in the pathogenesis of cardiovascular disease and heart failure in patients with type 2 diabetes, as increased levels of FGF-23 has also been associated with an increased risk of major adverse cardiovascular events including incident heart failure in this group of patients [9]. We used adenosine as a vasodilator substance, which primarily induces endothelium-independent vasodilation by acting directly on the vascular smooth muscle cells A_2_ receptors [21]. An independent continuous relationship between increased FGF-23 and impaired endothelial-dependent as well as endothelial-independent vasodilation have previously been reported in a large community-based study of subjects with normal renal function [22]. Our data support a similar relation between FGF-23 and vascular dysfunction in patients with type 2 diabetes, however, whether the effect of FGF-23 on the vasculature is through alterations in coronary microvascular function, promotion of vascular atherosclerotic calcification, or through physiological modifications in other steps of the vasodilatory process cannot be concluded from the present study. Reduced MPR—in the absence of obstructive coronary artery disease or other cardiovascular risk factors—is associated with heart failure with preserved systolic function [23], and, in the present cohort, we have previously demonstrated that both reduced stress MBF [14] and overall MPR [10] are associated with impaired diastolic function in patients with type 2 diabetes.

### FGF-23, LV mass and diastolic function

In rodents and in-vitro studies, FGF-23 has been demonstrated to directly influence cardiomyocytes and induce LV hypertrophy [24], one of the key characteristic of diabetic cardiomyopathy [25]. In our study, we found that LV mass was generally higher in our group of patients with FGF-23 above the median but we were not able to demonstrate a significant association between LV mass and FGF-23 on a continuous scale. This contrasts the findings of Mirza et al. [26] who showed an association between elevated FGF-23 and LV mass in a large cohort of patients examined with echocardiography. In their entire cohort, Mirza et al. found a relatively weak association between FGF-23 and LV mass which may have been mainly driven by patients with eGFR < 60 ml/min/1.73 m^2^, as they found the strongest association between FGF-23 and LV mass in this patient subgroup. We excluded patients with eGFR < 60 ml/min/1.73 m^2^ which may explain the discrepancy of the results, and why we were unable to demonstrate a significant association between the two variables on a continuous scale. In addition, we found that increasing levels of FGF-23 were associated with echocardiographic signs of impaired diastolic function. In diabetic cardiomyopathy, LV diastolic dysfunction has been suggested as the earliest functional change detectable and is almost always present prior to signs of LV systolic dysfunction [27]. Hypertension, ageing and diabetes are all associated with an increased risk of developing diastolic dysfunction [28–30], and recently it has been shown that higher glycemic variability may be associated with LV diastolic dysfunction independent of HbA1c [31]. Recent studies have shown, that treatment with SGLT-2 inhibitors dapagliflozin [32] or canagliflozin [33] can improve LV diastolic function in patients with type 2 diabetes, and that SGLT-2 inhibitors may play a future role in prevention and treatment of cardiovascular disease in patients with type 2 diabetes.

### FGF-23 and anti-diabetes therapy

In our study, we found no association between FGF-23 and treatment with SGLT-2 inhibitors, suggesting that the beneficial effect of SGLT-2 inhibitors on LV diastolic function may be through pathways independent of FGF-23. Insulin secretion, and increased plasma concentrations of insulin, is suggested to inhibit the formation of FGF-23 [34], which may explain why we found lower levels of FGF-23 in our patients treated with insulin. Furthermore, treatment with GLP-1 analogues alone and in combination with insulin were also associated with lower levels of circulating FGF-23. In the LEADER trial [35], treatment with GLP-1 analogues had a positive effect on a composite cardiovascular outcome consisting of cardiovascular death, nonfatal myocardial infarction and nonfatal stroke. The beneficial effect was evident in both patients with and without chronic kidney disease but did seem to be numerically greater in patients with eGFR < 60 ml/min/1.73 m^2^, who should also be expected to have the highest levels of circulating FGF-23. In patients with gestational diabetes, FGF-23 may be a promising indicator of subclinical arteriosclerosis [36], and FGF-23 has previously been shown to be positively correlated with the development of coronary atherosclerosis [37]. The anti-atherosclerotic effect of GLP-1 analogues has been suggested as an explanation for the reduction in cardiovascular morbidity and mortality associated with GLP-1 therapy [38], whereas very little data exists on how GLP-1 therapy affects the myocardial microcirculation. One small study (n = 24) of patients with type 2 diabetes and no history of coronary artery disease, treated for 10 weeks with the GLP-1 analogue Liraglutide, found no improvements in coronary flow reserve [39]. However, this study used transthoracic Doppler echocardiography to measure coronary flow reserve, limiting the sensitivity for detecting changes in myocardial microvascular function, especially in the relatively small sample size. In addition, the short follow-up period of only 10 weeks may have been insufficient for demonstrating a beneficial effect of GLP-1 therapy on coronary flow reserve. Therefore, to further investigate this topic, larger prospective studies with a longer follow-up period using a more sensitive measure for myocardial microvascular function are warranted. The observational design of our study precludes us from commenting on causality, however, with the apparent correlation between FGF-23 concentrations and treatment with GLP-1 analogues demonstrated in the present study, it would be of interest for future studies to investigate how GLP-1 therapy affects circulating levels of FGF-23 and its relation to cardiovascular outcome in a longitudinal setup.

## Limitations

Our study has some limitations that should be mentioned. The observational design of our study prohibits us from concluding on causality and, as such, our findings are only speculative and should be confirmed in prospective longitudinal studies before any definite conclusions can be made. We did not perform coronary angiography to exclude coronary artery disease and silent ischemia as this would have been both logistically difficult and ethically inappropriate in our population of asymptomatic patients. The stress perfusion CMR scan would have detected any significant ischemia, and as such we do not believe undetected coronary artery disease to have had an important impact on our findings. However, any non-significant coronary artery stenosis or an artery system with balanced ischemia could be a confounding factor as these would not necessarily be revealed from the CMR stress perfusion scan. We did not measure 1,25(OH)_2_-vitamin D_3_ or phosphate levels in our study cohort nor did we record information regarding self-administered or prescribed calcium and vitamin D supplements or dietary phosphate intake. As we have not been able to consider these variables in our calculations we cannot exclude these as a confounding factors.

## Conclusions

In conclusion, increased levels of circulating FGF-23, in patients with type 2 diabetes and normal to mildly impaired kidney function, is associated with impaired cardiac diastolic function and decreased myocardial perfusion reserve, caused by a decrease in maximal myocardial blood flow during pharmacological vasodilator stress. Peripheral neuropathy is associated with higher concentrations of circulating FGF-23, whereas patients treated with GLP-1 analogues or insulin have lower levels of circulating FGF-23. Our results support a relationship between FGF-23 and imaging markers of diabetic cardiomyopathy, and FGF-23 has previously been proposed as a potential predictor of cardiovascular disease in patients with diabetes [40]. These findings warrant for future studies to examine the predictive value of FGF-23 for cardiovascular morbidity and mortality in patients with type 2 diabetes, as FGF-23 may prove to be a useful biomarker for identifying patients at high risk of adverse cardiovascular outcome.

## Data Availability

The datasets analysed during the current study are not publicly available due consideration of intellectual property, due to continuing analyses by the study investigators but are available from the corresponding author on reasonable request.

## Abbreviations

ACE-i/ARB: Angiotensin converting enzyme inhibitors/angiotensin II-receptor blockers
CAD: Coronary artery disease
CMR: Cardiac magnetic resonance imaging
ECV: Extracellular volume
EDV: End-diastolic volume
eGFR: Estimated glomerular filtration rate
ESV: End-systolic volume
FGF-23: Fibroblast growth factor-23
GLP-1: Glucagon like peptide-1
LA: Left atrium
LGE: Late gadolinium enhancement
LV: Left ventricle
LVEF: LV ejection fraction
MBF: Myocardial blood flow
MPR: Myocardial perfusion reserve
NSR: Næstved/Slagelse/Ringsted
SGLT-2: Sodium glucose co-transporter-2
UACR: Urinary albumin/creatinine ratio
